# Preparation for pyeloplasty for ureteropelvic junction obstruction using a patient-specific laparoscopic simulator: a case report

**DOI:** 10.1186/1752-1947-6-338

**Published:** 2012-10-05

**Authors:** Hiroyuki Yamanaka, Kazuhide Makiyama, Tomoyuki Tatenuma, Ryoko Sakata, Futoshi Sano, Yoshinobu Kubota

**Affiliations:** 1 Department of Urology, Yokohama City University, 3-9 Fukuura, Kanazawa-ku, Kanagawa, Japan

**Keywords:** Surgical simulator, Ureteropelvic junction obstruction, Pyeloplasty, Laparoscopy

## Abstract

**Introduction:**

Training systems for laparoscopic surgery are useful for basic training but are not suitable for specific training corresponding to the condition of a given patient. We, therefore, have developed an unusual training system: a patient-specific simulator for laparoscopic surgery. When specific data of each individual patient are entered, this system helps surgeons perform a “rehearsal” operation. We applied this technique in laparoscopic surgery by using volume data obtained by multislice computed tomography imaging.

**Case presentation:**

A 39-year-old Japanese woman consulted a doctor because of back pain and underwent pyeloplasty after an examination revealed a ureteropelvic junction obstruction. Computed tomography data showed that the network of arteries and veins was very complicated. Therefore, we decided to use our simulator before performing surgery. Simulation was helpful because we could obtain information about the complicated vessel network and “rehearse” the procedure.

**Conclusions:**

Our simulator allows surgeons to perform a sham operation with different perspectives and tactile sensations and has received favorable reviews from users.

## Introduction

In recent years, laparoscopic surgery has attracted attention as a minimally invasive type of surgery because of the small size of surgical wounds and early recovery [1]. Teaching laparoscopic skills is a challenge in surgical training programs. Because of the highly technical nature and the steep learning curve, students and residents must acquire laparoscopic skills before performing laparoscopy in the operating room.

**Figure 1:**
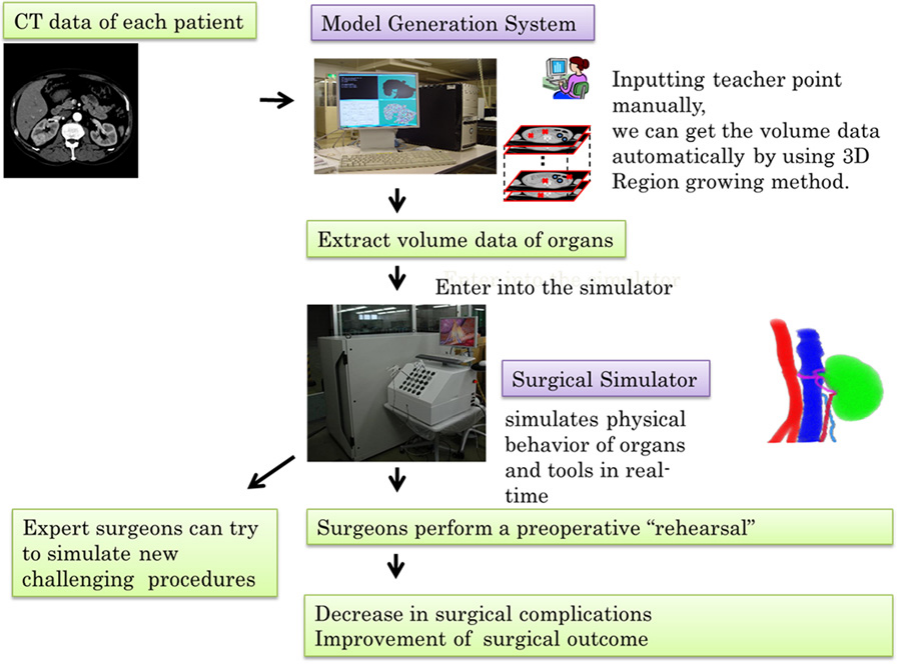
Specific data of each individual patient are entered into this system, which was designed to help surgeons perform a “rehearsal” operation.

The latest simulator can reproduce the entire procedure of laparoscopic radical nephrectomy [2,3]. Some training systems that simulate a surgical procedure are available commercially. They are useful for basic training but are not suitable for training surgeons to respond to the specific conditions of a given patient [2,4-6]. Unlike other surgeries, urological surgeries need a great deal of information about vessels, ureters, and tumors hidden by other organs; this protects the organs and shortens the operation time. In partial nephrectomy, both the laparoscopic and retroperitoneal views of renal cancer are observable and so the surgical procedure and the location of the trocars can be determined before the operation. In radical nephrectomy, the location of the trocars for a smooth operation can be seen on the simulator. We, therefore, have developed an unusual training system, a patient-specific virtual reality (PSVR) simulator for laparoscopic surgery and set our sights on its commercial potential [7]. Because specific data of each individual patient are entered into it, this system allows surgeons to perform a “rehearsal” operation. In addition, we use multislice computed tomography (CT) imaging technology in laparoscopic surgery. CT images of each patient who is scheduled to undergo surgery are transferred to the simulation system. The specific organ volume data of each patient are extracted on our simulator, and surgeons perform a pre-operative “rehearsal” (Figure 1). Some PSVR simulators have been reported and are in commercial use [8], but there is no PSVR simulator in urology.

We report a case of ureteropelvic junction obstruction (UPJO) in which we used our simulator. Laparoscopic pyeloplasty has developed worldwide as the first minimally invasive option to match the success rates of open pyeloplasty [9,10]. Success in this procedure requires a pre-operative examination of crossing vessels and the urinary tract. Simulation was helpful because the network of arteries and veins was very complicated in this case and we could “rehearse” the surgery.

## Case presentation

A 39-year-old Japanese woman consulted a doctor because of back pain, and UPJO was detected after an examination. She was referred to our department and agreed to undergo laparoscopic surgery. A renogram showed that the function of her left hydronephrotic kidney was maintained to some extent, and surgery was thought to be effective. However, CT data revealed that the network of arteries and veins was very complicated. Therefore, we decided to use our simulator before surgery, and our patient agreed.

**Figure 2:**
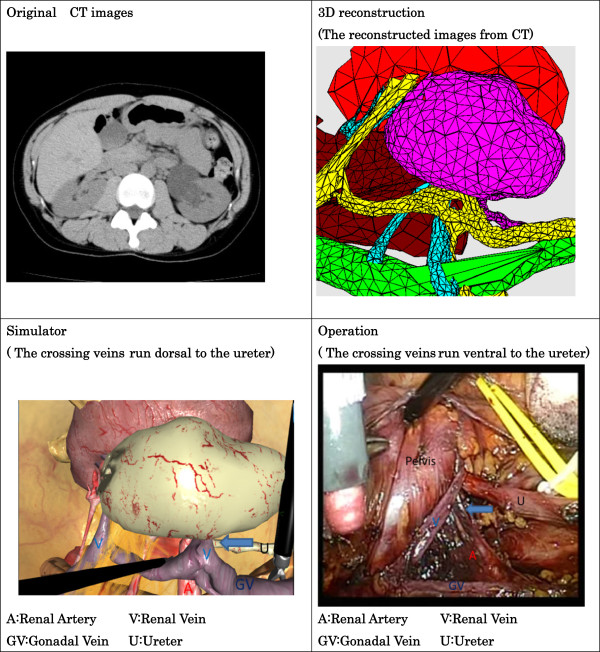
**The original computed tomography images were converted to three-dimensional images.** A comparison of the simulator and the operation is shown.

CT data were saved in the DICOM (Digital Imaging and Communications in Medicine) file format. CT was performed with slices of 1mm in thickness from the chest to the pelvis, and its DICOM file was reconstructed to obtain three-dimensional (3D) volume data. The reconstructed organs were shaped with 3D mesh by using computer graphic techniques. After 3D conversion, the data were transmitted to our simulator, and relations and locations of organs were set. Fibrotic tissue and fat were added appropriately around the organs, and reconstruction of the body was completed (Figure 2).

During the simulation, we first placed the camera and trocars on the body surface and obtained a virtual view of the abdominal cavity. When we changed the position of the trocar, the virtual view of the abdominal cavity also changed. Thus, we could choose a better position of the trocar in this pre-operative simulation. There were four renal arteries, two renal veins, and one gonadal vein that bifurcated in the middle. The lumbar vein was not important during surgery and therefore was not depicted.

During surgery, we chose the right flank position and four trocars. The pneumoperitoneum pressure was 10mmHg. We approached the retroperitoneum from the lateral aspect of the descending colon. We found the gonadal vein and ureter and then reached the renal hilum. We found an anonymous vein that tightened the ureter. We removed the target vein and ureteral stenosis and performed a dismembered pyeloplasty after placing a ureteral stent. The pathological finding was fibromuscular tissue, which was thought to be the cause of the UPJO. Operative and pathological findings showed that ureteral stenosis occurred not only because of the crossing vein but also because of endogenous stenosis. Our patient was discharged from our department without complications. After surgery, there were still four renal arteries, two renal veins, and one gonadal vein; the status of the lumber vein remained unknown because the lesion was outside the target area. The locations of the arteries, veins, ureter, and UPJO were almost the same. However, the anonymous vein, which tightened the ureter and ran dorsal to it on the simulator, ran ventral to it during surgery (Table 1).

**Table 1:** The main features of the simulator and the operation

**Features**	**Simulator**	**Operation**
Number of renal arteries	4	4
Number of renal veins	2	2
Number of gonadal veins	1 (forked in the middle of itself)	1 (forked in the middle of itself)
Number of lumber veins	Unknown (out of operation area)	Unknown (out of operation area)
Number of ureters	1	1
Lesion of obstruction	Ureteropelvic junction	Ureteropelvic junction
Impression of surgeon	A meaningful simulation	

## Discussion

Many commercially available laparoscopic simulators aim at the mastery of basic skills by practice with a programmed tutorial. These simulators can evaluate the basic skills by measuring the time or movement of forceps [2,11]. On the other hand, because our simulator uses specific data of each individual patient, it meets the demands of a trained doctor who wants to simulate a complicated surgery. In renal and ureteral surgeries, the network of arteries and veins is complicated and varies. Therefore, we should obtain detailed information and be well prepared to avoid confusion and complications during the surgery. In fact, the vascular structure is complicated and reconstruction of the 3D data in our UPJO case was very time-consuming. Therefore, there were some structural differences between the simulated and actual vascular networks.

The higher the resolution of the images, the more accurate the reconstructed 3D images. Some reports suggested that a slice thickness of about 2mm is required to construct clear 3D images [12,13]. At present, we are trying to combine the CT data (precontrast image, arterial phase, and nephrographic phase) and to improve the software so that tumors, vessel trees, or urinary ducts are highlighted in the simulation. (Now it can be depicted only in the software.) If we acquire more information about the patient, the precision of our simulator will improve. We are also trying to include the organ’s properties in the simulator so that it will provide a more realistic situation in the future.

By means of a patient-specific simulator, surgeries may be more accurate and proceed more smoothly because the surgeon has accurate anatomical information. In addition, it is possible that the time required for surgery, pre-operative risks, and complications will decrease. As we are finding from other clinical cases, surgeons could carry out meaningful pre-operative training and stated that the simulation was useful for constructing pre-operative images, although the operation time was not decreased. Trocar positions, which were simulated, were appropriate in other cases [7].

We need to validate the functionality of this simulator and improve the precision of the reconstructed 3D images before it can be made commercially available. However, we are cooperating with gynecology and general surgery departments to put our simulator to use, so we are confident that it will be the next-generation operative technology. (Our project is also available online [14].)

## Conclusions

We reported a case of UPJO in which we used our simulator to “rehearse” the surgery. We demonstrated that our simulator was useful and could solve some problems. Our simulator has been improved, and its completion is under way.

## Consent

Written informed consent was obtained from the patient for publication of this manuscript and accompanying images. A copy of the written consent is available for review by the Editor-in-Chief of this journal.

## Abbreviations

3D: Three-dimensional; CT: Computed tomography; DICOM: Digital Imaging and Communications in Medicine; PSVR: Patient-specific virtual reality; UPJO: Ureteropelvic junction obstruction.

## Competing interests

The authors declare that they have no competing interests.

## Authors’ contributions

All authors are clinicians from our urology department and treated our patient during her entire hospitalization. HY, KM, and YK are involved in the development of our simulator. All authors read and approved the final manuscript.
